# Influence of Stride Length on Pelvic–Trunk Separation and Proximal Plyometrics in Baseball Pitching

**DOI:** 10.3390/life15091440

**Published:** 2025-09-14

**Authors:** Dan K. Ramsey, Ryan L. Crotin

**Affiliations:** 1 Center for Doctoral Studies and Research, D’Youville University, Buffalo, NY 14201, USA; 2 ArmCare.com, Indialantic, FL 32903, USA; ryan@armcare.com; 3 Department of Kinesiology, Louisiana Tech University, Ruston, LA 71272, USA; 4 Sports Performance Research Institute New Zealand, Auckland University of Technology, Auckland 1010, New Zealand

**Keywords:** biomechanics, baseball pitching, stride length, pelvic–trunk counter rotation, elastic energy transfer

## Abstract

Pelvis and trunk counter-rotation are key factors known to effect throwing arm kinematics in baseball pitching, where energy or momentum is transferred from the lower extremities through to the trunk during the pitching cycle. The purpose of this study was to retrospectively analyze previously recorded motion capture data of 19 skilled competitive pitchers to test the a priori hypothesis whether different stride lengths affect transverse pelvis and trunk biomechanics. A blinded randomized crossover design was used where pitchers threw two simulated games at ±25% from desired stride length (DSL), respective of overstride (OS) and under-stride (US). Variables of interest included pelvic–trunk separation (PTS) angle or degree of uncoupling and proximal plyometric effect (PPE) or ratio between trunk–pelvis angular velocities, as surrogate measures of rotational and elastic energy transfer. Paired t-tests were used to compare across stride conditions. A one-way ANOVA with a Bonferroni post hoc analysis demonstrated stride lengths differed statistically, (DSL vs. OS *p* = 0.006), (DSL vs. US, *p* < 0.001), and (US vs. OS, *p* < 0.001). Despite the statistically different stride lengths, fastball velocities tracked with radar were consistent. No significant differences within and across innings pitched between OS and OS conditions were found. The ±25% stride length changes influenced temporal parameters within the pitching cycle. Shorter stride elicited by early SFC reduced time during the Generation phase and extended the Brace-Transfer duration (*p* < 0.001). Statistically different transverse pelvis and trunk kinematics at hallmark events and phases consequently influenced pelvic–trunk separation and proximal plyometrics. During the Generation (PKH-SFC) and Brace-Transfer (SFC-MER) phases, the pelvis and trunk were significantly more externally rotated (*p* < 0.001) with shorter strides, concomitant with less separation at the instant of SFC and the Generation phase with greater peak proximal plyometrics effect ratios peak during throwing arm acceleration, indicative of greater contribution of trunk angular velocity (*p* < 0.05). Greater transverse trunk angular velocities relative to the pelvis late in double support necessitates the throwing arm to “catch up” from a position of greater arm lag, which compromises the dynamic and passive stabilizers. In conclusion, stride length alters pitching biomechanics and timing of peak pelvic–trunk separation and trunk angular velocity relative to the pelvis. Increased shoulder and elbow tensile stress is to be expected, consequently increasing risk for injury.

## 1. Introduction

Baseball pitchers are at greater risk for overuse injury because they throw more in one game at higher metabolic cost than their teammates [1]. Fatigue, a term that is used ubiquitously, is a subjective surrogate to denote the decline in a person’s capacity to perform physical activity, often associated with increases in the real or perceived difficulty of a task or exercise [2,3]. However, it is important to differentiate the task-dependent nature of fatigue, peripheral and central [4]. Their etiology and perceptions differ. Peripheral fatigue arises from impairment of the muscles’ physiological processes, leading to reduced voluntary force-generating capacity during physical exercise, whereas central fatigue relates to the decreased capacity of the central nervous system, which decreases the neural drive to activate muscles [5]. Their determinants are interdependent and should not be considered in isolation. Therefore, fatigue in baseball must consider the multifactorial mechanisms involved. Intrinsic factors include pitchers’ roles (starter vs. reliever) and workloads (pitch count, innings pitched, pitch type (fastball vs. off-speed), and outings, as well as insufficient rest recovery between outings). Evidence suggests that pitching fatigued manifest kinematic and kinetic alterations that are often associated with performance decrements [6]. Analysis of ball velocity to infer onset of fatigue, however, is questionable given that altered stride length as a compensatory lower extremity mechanical response to prolonged physical exertion may potentially mask ball velocity decrements and go undetected in competition [7]. Consensus in the biomechanical literature suggests that fastball velocity on its own is not a strong predictor of elbow varus torque associated with UCL injuries [8].

Improper pitching biomechanics have long been attributed with increased stress to the throwing elbow and shoulder [9]. Throughout the baseball pitching cycle, movements progress up from the feet and lower extremities, through the trunk and throwing arm, and culminate at ball release. Previous findings suggest longer strides have a detrimental physiological cost yet may be beneficial mechanically in mitigating throwing arm stress, whereas shorter strides evoke inefficient pitching mechanics in preparing the throwing arm for maximal external shoulder rotation prior to ball release. Significant changes in physiological, momentum, performance and temporal profiles, ground reaction, and lower extremity dynamics have subsequently been reported, augmenting the initial findings [7,10,11,12,13,14,15,16,17,18]. The findings from each study were independent because they differ in their research question, hypothesis, analytic methods, and conclusions yet the findings are complementary in supporting how coordinated 3D lower extremity biomechanics are altered in response to changes in stride length, influencing the kinetic chain that potentially induces compensatory throwing mechanics and predisposes pitchers to greater risk for injury.

Pelvic and trunk counter-rotation are key factors in pitching, where energy or momentum is transferred from the lower extremities through to the throwing hand [19,20,21]. Trunk rotation along the longitudinal (vertical) axis involves rotations between the pelvis and upper torso [22]. Optimal sequencing between the pelvis and trunk allows for efficient transfer of energy through the kinetic chain, thereby improving ball velocity without increasing load on the shoulder and elbow [23,24]. Improper pelvic–trunk sequencing confounds pelvic–trunk separation [25] that causes breakdown in the kinetic chain and impedes momentum transfers to the throwing arm [23]. The concomitant increase in upper extremity joint loading is manifested by greater maximal shoulder external rotation angles, horizontal adduction lag, and dropped elbow with insufficient flexion, which predisposes pitchers to increased risk of shoulder and elbow injury [22,23]. Early onset of pelvic and trunk rotation was associated with affecting shoulder and elbow biomechanics, increased shoulder internal rotational and elbow valgus torques, and greater risk for surgery [19,20,26,27].

Pelvis–trunk separation is a key indicator of this kinetic chain function, as it represents the loading of lower extremity into a torsion spring through the core. The axial pelvic–trunk separation (PTS) angle, calculated by subtracting the trunk position from the pelvis position, is known to effect throwing arm kinematics. Perhaps altering stride length and drive leg propulsion influences axial PTS, which may increase the risk of throwing arm injury. Therefore, this retrospective analysis investigated stride length influences on (i) transverse PTS and (ii) sequencing (trunk-to-pelvis transverse angular velocity ratio, otherwise known as the proximal plyometric effect (PPE)) during fastball pitching. Altered stride length and drive leg propulsion [12] could influence axial PTS, which may increase risk of throwing arm injury.

This retrospective analysis centers on the a priori hypotheses that stride length influences (i) transverse PTS and (ii) sequencing (trunk-to-pelvis transverse angular velocity ratio, otherwise known as the proximal plyometric effect (PPE)) during fastball pitching to augment previous findings. As a result, throwing arm injury risks can be exacerbated by stride length compensation.

## 2. Materials and Methods

This study was part of a comprehensive single cohort full-body biomechanical analysis of healthy and skilled competitive pitchers from collegiate and high school seasonal travel programs from across Western New York [10]. The purpose was to investigate how coordinated lower extremity biomechanics are altered in response to changes in stride length, influencing the link-segment-model and kinetic chain that potentially induce compensatory overhand throwing mechanics in baseball pitching delivery, which predisposes players to increased risk of throwing arm injury. The scope of this investigation focused on testing the a priori hypothesis that ±25% changes from desired stride length, respective of over-stride (OS) and under-stride (US), impacted PTS angle and PPE.

### 2.1. Experimental Approach

This research was a retrospective analysis of previously collected and post-processed motion capture data from the original single cohort of 19 healthy and skilled competitive pitchers from collegiate and high school seasonal travel programs from across Western New York [10]. The scope of this investigation focused on testing the a priori hypothesis that ±25% changes from desired stride length, respective of over-stride (OS) and under-stride (US), impacted PTS angle and PPE. The protocols for subject recruitment, motion capture, and data post-processing have been published previously elsewhere [7,10,11,12,13,14,15,16,17,18]. Testing was undertaken in-season from April to September during the daytime at the Biomechanics Laboratory of Human Movement at the University at Buffalo.

### 2.2. Participants

In brief, nineteen healthy and skilled competitive pitchers (aged 18.63 ± 1.67 years, height 1.84 ± 0.054 m, mass 82.14 ± 0.054 kg) from collegiate and high school seasonal travel programs from across Western New York participated. Fifteen threw right-handed and four with their left hand. All competed at least one year prior, with no previous arm injuries within two years prior to the study. Anyone with previous injury or insufficient competitive experience was excluded. Each gave informed consent, or parental consent was granted for minors in accordance with the Children and Youth Institutional Review Board of the University at Buffalo.

### 2.3. Experimental Design

**Figure 1:**
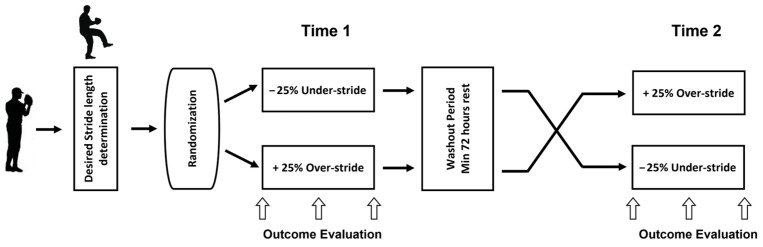
Two-sequence crossover design (AB/BA) separated by a washout period of 72 h rest.

A blinded randomized within-group crossover time series design was utilized, where pitchers threw 2 simulated games at ±25% changes from desired stride length. Participants were randomly assigned using permuted-block randomization and stratified by intervention to commence with either the over-stride or under-stride sequence. After the 72-hour rest washout period, the pitchers were crossed over to the alternate condition (Figure 1).

### 2.4. Data Collection

**Figure 2:**
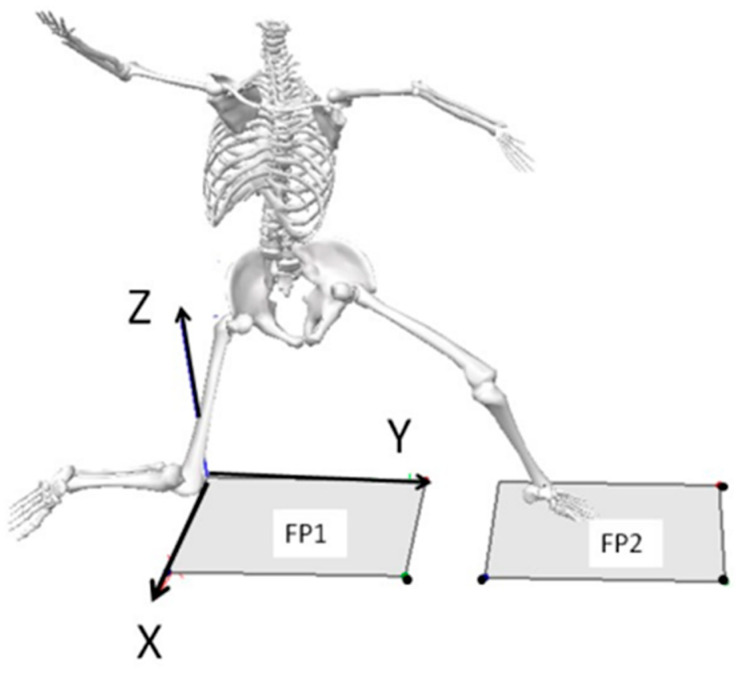
A right-handed orthogonal global cartesian (X-Y-Z) coordinate system aligned with two integrated force plates (FP1 and FP2) with the horizontal forward y-axis in the direction of the pitch towards home plate was used to describe pitching in 3D in space.

Biomechanical assessment involved conventional 3D motion capture where whole-body motion was tracked using an integrated 8-camera motion capture system (MX20-Vicon Motion Systems, Centennial, CO, USA) synchronized with two floor embedded force plates (KISTLER Corp., Amherst, NY, USA) that were aligned in series so that drive and stride legs contacted opposing force platforms to track pitching delivery. Retroreflective markers were affixed bilaterally to the whole body and reflective tape was secured to the baseball to track whole-body motion and the instant of ball release. Respective baseball velocities were obtained with a radar gun and LED display (Jugs Sports, Tualatin, OR, USA). Motion capture and force plate data were sampled at 240 Hz and 960 Hz, respectively, whereas the radar gun provided instantaneous feedback to encourage that fastballs be thrown maximally. Marker trajectories and times series data were abstracted and performance indices computed. The global coordinate system was referenced by +X (mediolateral axis in the direction of third base), +Y (axis in the direction of home plate), and +Z (vertical axis), as shown in Figure 2.

### 2.5. Stride Length Determination

After a standardized 10 min full-body general warm-up, each participant threw 30–40 practice balls (Rawlings Group, St. Louis, MO, USA) directly toward a 6.5′ × 6′ catch net (Rawlings Group, St. Louis, MO, USA), distanced at 5.69 m owing to lab constraints rather than from the standard 18.4 m, and with the radar gun positioned behind the net at a height of 1.02 m to best track ball velocity. Ball velocity was relayed to the pitcher via an LED display for instantaneous feedback to ensure fastballs were thrown maximally. The pitching delivery was standardized to the traditional stretch delivery with throwing intensity progressively increased to 100% effort until the 20th delivery. It is believed that by controlling pitching from a consistent stretch position enables pitchers to utilize less unnecessary movement over time, allowing for consistency for data comparisons.

Motion recordings were recorded (Vicon Nexus 1.8, Oxford Metrics, Denver, CO, USA) and ball velocities tracked while throwing at 100% effort between the 20th to the 25th delivery, at one’s respective desired stride length. The trials with the two fastest overhand throws between the 20th and 25th warm-up throws were selected as the pitcher’s own desired stride length. Stride leg peak knee height was depicted at the highest vertical displacement of the supra-patellar marker during the wind-up. Desired stride length (DSL) was calculated as the mean horizontal distance between the drive foot calcaneus at a peak knee height to the stride foot calcaneus at stride foot contact. Ground reaction force for both drive and stride legs were normalized to body weight, from which SFC was determined when the leading (stride) foot contacted the second (opposing) force plate and registered vertical ground reaction force that exceeded 5% body weight. Thereafter, desired stride length was manipulated by either increasing or decreasing stride by ±25% (determined a priori), which represents respective over-stride (OS) and under-stride (US) pitching conditions to challenge throwing mechanics. The remaining practice throwing trials were used to acclimatize to the stride condition. All stride lengths (DSL, OS, and US) were expressed in meters (m) and normalized to percent body height (BH%).

Areas over the force platforms were marked to indicate drive foot and stride foot placement for both OS and US conditions, where participants were encouraged to target during the simulated games. Ample warm-up throwing prior to motion recordings was provided to acclimatize pitchers to the OS or US conditions. During simulated game play, twenty overarm throws at 100% effort were completed per inning at a ratio of 3 fastballs to 1 change-up. Approximately 15 s rest was allocated between throwing bouts with 9 min rest prescribed between innings. Five warm-up throws were allocated before each inning. Testing ceased after the 80th delivery. In total, each pitcher threw approximately 130 times per simulated game.

### 2.6. Data Management and Post-Processing of Kinematic and Kinetic Data

Visual 3D software (Visual 3D v4.9, C-Motion Inc., Rockville, MD, USA) was used for post-processing kinematic and kinetic data. Marker trajectories and ground reaction force data were filtered using a 4th order dual-pass Butterworth filter at 13.4 Hz and 40 Hz, respectively [28,29,30,31].

### 2.7. Calculations

**Figure 3:**
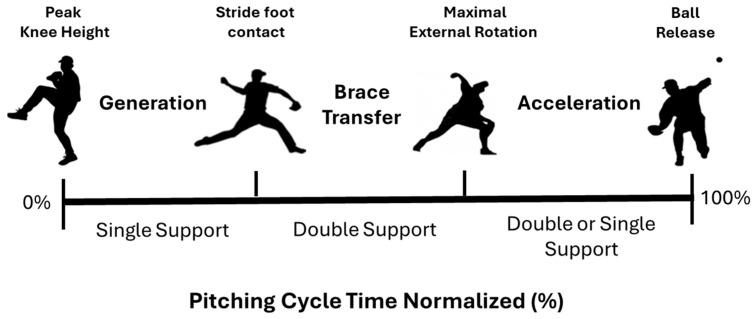
Important hallmark events (PKH, SFC, MER, and BR) and phases (GEN, BT, and ACC) depicted in the time normalized pitching cycle.

Overhand throwing was captured from peak knee height in the delivery through to ball release, as shown in Figure 3. Specific spatiotemporal parameters identified from kinematic data were used to indicate specific hallmark events and time normalize the pitching cycle to 100%, with peak knee height (PKH) coincident with 0% and ball release (BR) terminating at 100% (identified by visual inspection of ball and hand marker trajectories when the distance between the hand marker and reflective baseball increased 1 cm). Other hallmark events included stride foot contact (SFC) and maximal external shoulder rotation (MER) and three phases were defined between hallmark events:

(i) Generation (GEN) Phase: From PKH to SFC.
(ii) Brace-Transfer (BT) Phase: Between SFC and MER.
(iii) Acceleration (ACC) Phase: From MER to BR.

**Figure 4:**
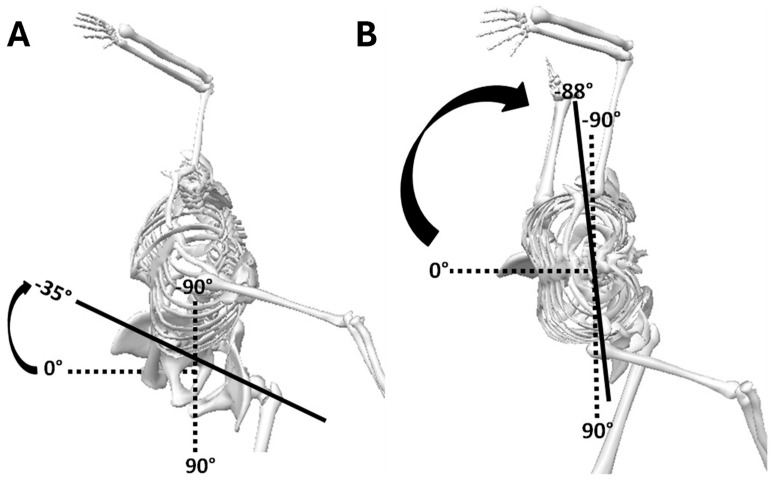
Illustration of how pelvis and trunk kinematic data were calculated along the vertical *z*-axis. (**A**) Pelvis rotation and (**B**) trunk rotation. Axial pelvic and trunk rotation kinematics were considered internal (+) or moving toward home plate and external (−) or away from home plate.

Axial pelvic rotation was denoted as the angle between a line connecting the 2 anterior superior iliac spines (interiliac line) relative to the sagittal (leading laboratory axis) or pitch direction toward home plate [21,32], whereas for upper trunk rotation, the angle was between the pelvis and upper trunk (Figure 4). Pelvic rotation at 0° was considered when the pelvis faced home plate or was perpendicular with the leading laboratory *y*-axis, and −90° when the pelvis was aligned with the pitch direction toward home plate.

Separation angles between the trunk and pelvis were calculated throughout the entire pitching cycle, referencing pelvic position to the trunk. Mathematical formulas were used as follows:

- Pelvic–trunk Separation Angle (PTS) = Pelvis θ − Trunk θ
- Proximal Plyometric Effect (PPE) = Trunk ω/Pelvis ω

Pelvic–trunk separation angle ahead of the trunk (pelvis rotated more than the upper trunk toward the pitch direction) was considered positive (+) and negative (−) when pelvic separation was behind the trunk.

### 2.8. Statistical Analysis

Statistical analyses were performed using SPSS 19 (SPSS Inc., Chicago, IL, USA) utilizing a series of planned paired t-tests for comparisons of PTS and PPE ratios, which were performed to analyze the presence of a difference between the two stride conditions at hallmark events and phases. Statistical significance, determined a priori, was set at *p* < 0.05 for all statistical tests.

## 3. Results

Ball velocity trends during games are commonly used to assess exertion and performance, where fatigue is thought to be associated with increased workload (number of innings and throws) and diminished peak ball velocity. As reported elsewhere, ball velocity performance indices remained unchanged over innings accrued (mean fastball velocity 123.5 ± 7.98 km/h vs. 122.7 ± 7.19 km/h and mean change-up velocity 109.1 ± 10.1 km/h vs. 106 ± 9.09 km/h) despite the ±25% stride adjustment, indicating the potential for compensation of respective over- and under-stride conditions [7,18]. However, fatigue may not always present as ball velocity decrements because of compensatory throwing biomechanics’ aid in maintaining peak ball velocity.

**Figure 5:**
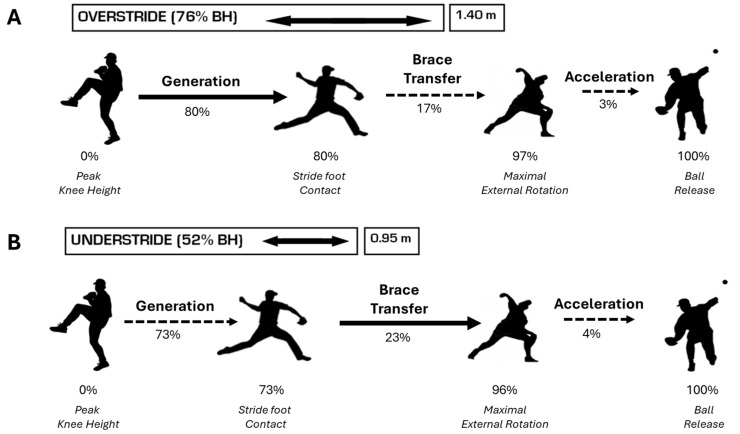
Stride length affected the instant of stride foot contact. Respective stride lengths relative to body height were 76% for OS and 52% during US. (**A**) Longer strides showed a later instant of SFC, longer GEN, and shorter BT. (**B**) Shorter strides demonstrated an earlier SFC, shorter GEN, and longer BT. The acceleration phase between stride conditions was no different. Despite temporal differences, ball velocities remained unchanged.

Respective mean stride lengths (m) and (% BH) differed statistically, DSL vs. OS (*p* = 0.006), DSL vs. US, (*p* < 0.001), and US vs. OS, (*p* < 0.001). For OS (+25% DSL) measured 1.40 ± 0.15 m, 0.76 ± 0.07%BH, DSL (measured during warm-up) was 1.24 ± 0.17 m, 0.67 ± 0.09%BH, and US (−25% DSL) reportedly 0.95 ± 0.14 m, 0.52 ± 0.08%BH/ [7,18]. The 24% difference between OS and US, between 50 and 80% body height as shown in Figure 5, required pitchers to adjust their throwing mechanics.

Table 1 and Figure 5 depict the out of phase timing of pitching events relative to stride length normalized to percent time. The time from PKH-SFC (Generation) was statistically longer for OS and significantly shorter from SFC–MER (Brace-Transfer) compared to US. MER and BR were not different between groups, indicating equivalent response times for the throwing arm.

Transverse Plane Kinematics

Greater pelvic internal rotation throughout GEN (*p* < 0.001), as well as during BT (*p* < 0.05), corresponded with increased angular velocities during GEN (*p* < 0.001) with longer strides (Table 2). Similarly, trunk internal rotation at GEN (*p* < 0.001) and SFC (*p* < 0.05) and during BT (*p* < 0.05) were greater for increased strides. Internal rotation velocity for the trunk was greater for increased strides during GEN (*p* < 0.001) and at SFC (*p* < 0.05). Shorter strides revealed greater maximal pelvic angular velocity (*p* < 0.001) after SFC and greater magnitudes of internal trunk rotation at MER (*p* < 0.05).

Peak internal trunk rotation velocities were statistically higher (*p* < 0.05) with the shorter strides, which occurred at 90% in the pitching cycle or close in proximity to MER (Table 3). Over-stride revealed greater pelvic and trunk internal rotation during GEN, at SFC, and during BT. GEN internal rotation velocities for the pelvis and trunk were greatest for over-stride. Under-stride indicated higher internal rotation velocity at MER. Overall peak internal rotation velocity for the pelvis was greater for under-stride than over-stride. No significant differences in peaks were found for the trunk.

Concerning the events from Table 4, at PKH, separation angles increased with US, which is indicative of greater transverse pelvic counter-rotation relative to the trunk, which rotates in the opposite direction, away from home plate (*p* < 0.05). With OS, the pelvis was positioned further ahead of the trunk, revealing greater separation earlier at SFC (*p* < 0.05) and during GEN (*p* < 0.001). Although peak separation magnitudes during the Brace-Transfer phase were no different between stride conditions, onset occurred at 4% in the pitching cycle (immediately after SFC) with OS and later at 12% with US, well after SFC. Despite equivalent peak separation angles, greater proximal plyometric effects were observed during BT (*p* < 0.05) with OS, whereas US demonstrated greater effect throughout ACC (*p* < 0.05), with greater peaks evident at BR (*p* < 0.05).

**Figure 6:**
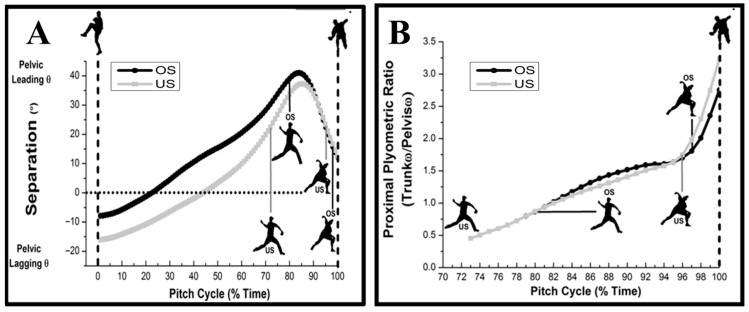
A comparison of (**A**) pelvic–trunk separation (PTS) and (**B**) proximal plyometric effect (PPE) profiles between under-stride (US) and over-stride (OS) conditions. Pelvic separation behind trunk (−) and pelvic separation ahead of trunk (+). Timing of peak separation was coincident for OS and US occurring in proximity to 85% of pitching cycle. Greater proximal plyometric effects evident with US during acceleration phase (MER to BR), where peaks occurred at BR for both conditions.

Pelvic–trunk separation (PTS) and proximal plyometric effects (PPE) were both affected by the ±25% changes in stride length (Table 5 and Figure 6). Higher separation angles (-) were observed at PKH with US (*p* < 0.05), whereas pelvis position with OS was further ahead of the trunk, with greater separation (+) observed during the GEN phase (*p* < 0.001) and at SFC (*p* < 0.05). Proximal plyometric effects were higher with OS during BT (*p* < 0.05), whereas US evoked greater effect throughout ACC (*p* < 0.05), with greater peaks evident at BR (*p* < 0.05).

## 4. Discussion

The aim of the study was to show how pitchers when altering their stride length influence transverse pelvis and trunk kinematics, thereby impacting the inter-relationship between pelvic–trunk separation and proximal plyometrics. Because the ±25% stride length significantly influenced timing at stride foot contact, at 73% and 80% in the time normalized pitching cycle between US and OS conditions, respectively [14], coordinated trunk and pelvic rotation was subsequently impacted. While stride lengths (% body height) at various competitive levels have been shown to vary between 70 and 90% [33], they have been described to be as low as 66% in less experienced athletes [34]. The 52% and 76% measured magnitudes for US and OS fall within these ranges, but the differences may be at least partially attributable to methodological differences.

Compensated ±25% stride lengths influenced pelvic trunk biomechanics, evident by changes in global pelvic and trunk kinematics at hallmark events (peak magnitudes at PKH, SFC, MER, and BR) and their respective onsets within the corresponding phases of the pitching cycle (GEN, BT, and ACC). Pitching with longer strides saw overall proximal segmental kinematics begin earlier in the pitching cycle, with significantly higher magnitudes were observed. Peak transverse angular velocities were also significantly different between stride conditions. Consequently, transverse pelvic–trunk kinematics during GEN (from PKH to SFC) was influenced by stride length, evident by greater internal rotation kinematics (rotation toward home plate) and earlier onset in the pitching cycle with OS, whereas pelvic–trunk internal rotation increased and started later with US, near MER.

Proximal internal rotations at SFC were significantly higher with OS while pelvic–trunk displacements were no different between conditions (Table 2). The increased displacements at SFC resulted in internal trunk rotation velocities nearly 200 °/s faster with the longer strides, whereas US saw trunk angular velocities increase to 450 °/s during the Brace-Transfer phase (SFC to MER) and achieved greater peak internal rotation velocities at MER. Thereafter, during the ACC phase (prior to BR), internal rotation displacements were similar between conditions (Table 2). Because the shorter stride lengths contributed to increasing BT duration, this provided ample time for transverse pelvis and trunk acceleration. Greater internal pelvic–trunk rotation following SFC may be to compensate for reduced total body forward momentum during generation from PKH to SFC [13,15]. Perhaps greater peak pelvic and trunk internal rotation velocities from Table 3 expressed later in the delivery when pitching with shorter stride lengths are the underlying mechanics responsible for the equivalent peak ball velocities being observed between conditions [7]. The increased transverse pelvic–trunk mechanics elicited by shorter stride lengths may mediate increased throwing arm kinetics, which predispose the elbow and shoulder to increased risk for injury. By increasing pelvic internal rotation range of motion at SFC, throwing arm kinetics may be reduced [20,21]. Others reported that transverse trunk kinematics may influence internal elbow valgus moments, as evidenced by the early onset of internal trunk rotations (prior to SFC), which correlated with increased elbow valgus moments [27,35]. In light of these findings, our results appear contradictory. On the one hand, overstride corresponds with the early onset of internal pelvic rotation and trunk rotation (prior to SFC), yet it has been associated with respective decreases [20] and increasing throwing arm kinetics [27]. The shorter strides, however, resulted in greater pelvic and trunk rotation away from home plate following SFC, which, respectively, exacerbates or decreases throwing arm kinetics [20,27].

When transverse trunk kinematics further throwing arm horizontal abduction, the forearm “lags” further behind the upper arm as a result of its inertia, which is considered the primary mechanism responsible for increasing shoulder and elbow kinetics [19,36]. Throwing arm “lag” relative to stride length variation may best be interpreted through transverse momentum analyses by examining throwing arm transverse angular momentum relative to total body momentum (reflecting trunk momentum) [13,15,37]. The summed products of throwing arm segments’ angular velocities and transverse rotational inertias depict the overall throwing arm momentum, which is known to be affected by horizontal abduction velocity [37]. Horizontal abductor velocities, when affected by internal trunk rotation velocities, demonstrate variation in trunk–throwing arm momentum transfers by impacting momentum proportionality between the throwing arm and trunk [13,15]. As evidenced by momentum compensation ratios (MCRs), momentum proportions in the transverse plane during BT were affected by stride length, where greater MCRs with longer strides were observed, exemplifying a greater percentage of total body momentum being occupied by the throwing arm [13,15]. Reduced trunk transverse momentum relative to the throwing arm depicts reduced throwing arm “lag”, which is thought to aid in advancing the throwing arm toward home plate when pitching with longer strides [13,15]. Shorter strides demonstrated greater peak rates of internal trunk rotation and lower MCR, with no improvement in ball velocity [13,15]. Consequently, pitchers with short strides reduce throwing arm momentum proportions relative to the trunk, which may be indicative of inefficient and potentially injurious momentum transfers prior to acceleration [19,38], whereby increased throwing arm “lag” and kinetics are expected in relation to greater transverse trunk and pelvic velocities [19,20,27,39].

Pelvic–trunk separation and proximal plyometric effects were both affected by changes in stride length. Longer strides resulted in greater separation (pelvis and trunk counter rotate in opposite directions), which occurred earlier in the pitching delivery, with peaks within 4% time from SFC. Conversely, shorter strides revealed peak pelvic–trunk separation at approximately 12% after SFC. The timing of peak separation is critical. Near SFC allows for efficient energy transfer to the throwing arm. Despite non-significant pelvic–trunk separation magnitudes, greater pelvic internal rotation ahead of the trunk during GEN prepared peak separation occurred near SFC with longer strides. Early pelvic–trunk separation mediated greater proximal plyometric effect ratios earlier in pitching delivery (SFC to MER), which may regulate trunk angular velocity relative to the pelvis at BR. Shorter strides resulted in pelvic–trunk separation to occur later along with a greater than 3-fold increase in the trunk to pelvic angular velocity ratio at BR, denoting a greater peak proximal plyometric effect with reduced strides. High PPE ratios late in the pitching cycle may be to compensate for the reduced drive leg propulsion observed during the generation phase (peak knee height to stride foot contact) [12].

Pelvic–trunk separation, known as the “Serape Effect”, refers to the diagonal orientation of the abdominal core musculature, involving the rhomboids, serratus anterior, and internal and external obliques linking the left shoulder to the right hip and vice versa [40,41]. The leading (stride) hip opposite the throwing shoulder initiates the stretch-shortening cycle, the proximal plyometric effect, allowing the core musculature to produce greater force and power after rapid pre-stretch that assists throwing arm acceleration [28]. Proximal plyometric effect ratios may be useful to evaluate contralateral stretch-shortening across core musculature, where augmented trunk angular velocity with respect to the pelvis later in the pitching cycle may determine an adaptive change in lower body biomechanics, such as a reduction in stride length.

In the context of pitching delivery, perhaps altering stride influences coordinated throwing-side oblique muscle and contralateral (opposite side) pelvic internal rotators, which is integral for generating power. These muscles work synergistically to transfer energy from the lower body to the upper body, ultimately contributing to the speed and effectiveness of the throw. Increased trunk angular velocities were evident by the higher proximal plyometric effect ratios during ACC and Br with US, which is thought to compensate for the shortened GEN phase and be augmented by the extended propulsive efforts during the Brace-Transfer phase (double support as seen with short stride pitching). Greater pelvic external rotation at SFC (latent peak pelvic–trunk separation) and increased trunk internal rotation (higher proximal plyometric effect during ACC) were evident during US. Higher angular transverse trunk velocities during ACC have been associated with increased shoulder and elbow centrifugal forces, which is thought to be mediated by the throwing arm “lagging” further behind because of increased horizontal shoulder abduction and forearm rotational inertia [42]. Increasing throwing arm lag is thought to exacerbate inter-segmental joint distraction and has been implicated in a variety of throwing arm pathologies [42]. Latent pelvic rotation and delayed pelvic and trunk peak angular velocities are also known injury risk factors shown to increase shoulder and elbow kinetics, which supports the need to ascertain safety ranges in stride length to prevent unsafe pelvic–trunk kinematics [20,27,39].

**Considerations and limitations**

Stride length is pitcher-specific and fluctuates as games progress and throughout the season owing to many factors that can either be related to orthopedics, pitch type, physiologic, or central/peripheral fatigue. While only fastballs were assessed for this study, other pitch types may have slight differences in kinematic variables, thus decreasing the generalizability of these results to pitches other than the fastball.

Stride length has been shown to vary between 50 and 80% body height among collegiate and professional pitching populations [28,31] and by as much as 3.5% body height between fastball and curveball deliveries [43,44]. Therefore, this interval was integrated in this research design, where manipulating desired stride length by ±25% body height was determined a priori. The intent was to ensure a 5% difference in stride (normalized to body height) challenged throwing mechanics, whereby consequent compensatory biomechanical adaptations can be examined, which align with previous studies. Individual desired stride lengths were determined during the warm-up that preceded the simulated game conditions. The 25% difference ensured a minimum six-inch difference from the desired stride length in both directions and fell within this range.

The order in which participants were assigned to throw at ±25% desired stride length was randomized. A simple AB crossover design (common in early phase studies) was utilized because the advantage is that each subject serves as their own control, a smaller number of participants are required, and outcome responses can be contrasted with greater precision given the same number of subjects. The 72-hour washout period adhered to Major League Baseball’s safety protocols for 60 or more accumulated pitches per day. Instantaneous radar velocity feedback (Jugs Sports, Tualatin, OR) was used as a control to ensure balls were consistently thrown maximally.

The disadvantage was the inability to compare desired stride length data with the ±25% stride conditions. To enable comparison with desired stride length, more complicated crossover designs are necessary, perhaps the same desired stride length condition being administered in multiple different periods (e.g., ABAC or ABCABC designs) or to deploy a parallel-group design (ABC) requiring larger samples. In light of the fact that different profiles were evident, this may serve as the basis for future investigators utilizing more complex research design methods to substantiate these kinetic changes with desired stride length profiles.

The absence of a pitching mound may question the validity of the study, yet stride length has been shown to be relatively consistent among skilled amateurs when throwing from a mound or level ground [45,46]. Evidence suggests that throwing from a level surface may increase injury risk (throwing arm kinetics) especially at longer distances [45] and therefore we believe this research paradigm is relevant for examining kinematic and kinetic characteristics that may be considered important in preventing injuries. Pelvic kinematics and trunk angular displacements are consistent with previous mound studies with similar pelvic–trunk separation angles [28,39,43,45]. Notably, peak ball velocities between the mound and the results were equivalent, which strengthens the applicability of the flat ground model to describe stride length impacts on compensatory adaptations [19,28,37,39,40,41,44].

Because this study is part of a comprehensive single cohort full-body biomechanical analysis, the findings are complementary in that stride length may be an important contributor during the pitching delivery, where coordinated 3D lower extremity biomechanics are altered in response to changes in stride length influencing the kinetic chain. Power-flow up the link-segment-model, which is thought to regulate angular joint mechanics through to the trunk, shoulder, elbow, wrist, and hand, may potentially predispose players to increased risk of throwing arm injury. These findings have implications on training, performance, and throwing arm injury prevention.

**Practical applications:**

- Peak stretch-shortening of the intrinsic trunk internal rotators, reflective of maximal pelvic–trunk separation, is critical for high-power rotational movements like baseball pitching.
- The “peak proximal plyometric effect ratio”, based on sequenced transverse angular velocities between the trunk and pelvis, denotes the proportion of the trunk’s transverse angular velocity relative to the pelvis (rotational movement energy transfer) at the same instant in time.
- Peak pelvic–trunk separation was delayed with shorter strides. With less time spent during the generation phase (PKH to SFC), the pelvis is more externally rotated concomitant with less separation at the instant of SFC, which delayed peak separation in double support. Delayed peak separation timing increased peak proximal plyometrics effect ratios during throwing arm acceleration. Greater transverse trunk angular velocities relative to the pelvis late in double support necessitates the throwing arm to “catch up” from a position of greater arm lag, which compromises the dynamic and passive stabilizers. Increased shoulder and elbow tensile stress is to be expected, consequently increasing risk for injury.

## 5. Conclusions

Stride length regulates timing at stride foot contact that subsequently effected coordinated trunk and pelvic rotation and trunk–pelvis angular velocity ratios, both essential for efficient energy transfer from the lower extremities through the trunk to the upper body. During under-stride pitching, pelvic trunk separation was closed throughout the generation phase (PKH-SFC). Improper kinematic sequencing restricts stretching the core muscles, which is vital for storing and releasing elastic energy, consequently requiring greater trunk–pelvis angular velocity ratios later in the delivery. This compensation appears to increase arm lag and transverse trunk velocities later in the delivery, where the arm is forced to accelerate more rapidly to compensate for lost rotational energy from the core. This may elevate stress on the shoulder and elbow. In contrast, longer strides mediated more open pelvic–trunk separation in the pitching delivery, which better regulates proximal angular velocity ratios and energy storage, suggesting a more efficient transfer of force. However, the optimal degree of separation and proximal plyometrics remains underexplored for throwing health, which underscores the need for further research on stride mechanics, rotational compensation, and throwing arm biomechanics.

## Figures and Tables

**Table 1:** Stride length compensation effecting normalized timing of phase.

Stride Length	Generation PKH to SFC (% Time)	Brace-Transfer SFC to MER (% Time)	Acceleration MER to BR (% Time)
Over-stride	79.95 (2.79) **	16.95 (3.18) **	3.10 (1.39)
Under-stride	73.25 (4.68)	23.20 (4.84)	3.55 (1.46)

Mean (SD) for normalized times. PKH, Peak Knee Height; SFC, Stride Foot Contact; MER, Maximal External Rotation; BR, Ball Release; GEN, Generation Phase (PKH-SFC); BT, Brace-Transfer Phase (SFC-MER); ACC, Acceleration Phase (MER-BR). Significant differences (*p* < 0.001) **.

**Table 2:** Transverse kinematics.

		PKH	SFC	MER	BR	GEN	BT	ACC
Pelvis Angle (°)	Over-stride	−115.9 (36.5)	−61.0 ** (21.1)	1.74 (7.53)	7.79 (7.87)	−103.6 ** (16.1)	−27.3 * (19.8)	5.87 (1.79)
	Under-stride	−112.7 (38.8)	−85.2 (15.0)	1.88 (12.1)	10.7 (10.7)	−110.9 (9.63)	−44.5 (28.2)	7.72 (2.71)
Pelvis Velocity (°/s)	Over-stride	−13.8 (35.5)	417.7 (90.1)	283.1 (120.2)	92.7 (92.7)	118.8 ** (88.9)	461.6 (71.3)	157.6 (58.6)
	Under-stride	9.44 (56.4)	302.6 (166.9)	357.4 (146.1)	95.0 (126.2)	73.3 (53.7)	506.0 (99.0)	203.1 (93.0)
Trunk Angle (°)	Over-stride	−108.7 (27.6)	−99.9 * (25.3)	−15.8 (7.82)	−2.96 (6.28)	−114.8 ** (3.41)	−60.7 * (27.4)	−7.29 (3.95)
	Under-stride	−106.9 (28.5)	−116.5 (13.1)	−17.8 (12.4)	2.02 (8.93)	−116.5 (1.67)	−79.9 (31.6)	−5.45 (6.42)
Trunk Velocity (°/s)	Over-stride	−13.7 (30.7)	334.4 * (191.9)	474.9 * (105.3)	255.1 (86.6)	30.3 ** (64.0)	621.2 (123.9)	333.7 (67.1)
	Under-stride	−9.94 (38.5)	141.9 (185.1)	601.3 (149.3)	307.3 (114.4)	4.46 (20.5)	562.8 (235.4)	444.3 (105.0)

Mean (SD) for internal (+) and external (−) rotation displacements (degrees) and angular velocities (degrees/sec) of pelvis and trunk at normalized events and phases. Significant differences indicated (*p* < 0.001) ** and (*p* < 0.05) *. PKH, Peak Knee Height; SFC, Stride Foot Contact; MER, Maximal External Rotation; BR, Ball Release; GEN, Generation Phase; BT, Brace-Transfer Phase; ACC, Acceleration Phase.

**Table 3:** Peak transverse kinematics.

		Pelvis Internal Rotation	% Time		Trunk Internal Rotation	% Time
Peak Angular Velocity (°/s)	Over-stride	584.1 ** (62.7)	85.4 (8.71)	Over-stride	797.0 * (82.5)	89.8 (3.78)
	Under-stride	658.9 (73.3)	87.5 (3.47)	Under-stride	851.0 (94.2)	90.3 (2.43)

Peak angular velocities and normalized time at peaks for pelvic and trunk internal rotation (+). Significant differences indicated (*p* < 0.001) ** and (*p* < 0.05) *.

**Table 4:** Pelvic–trunk separation and proximal plyometric effect.

		PKH	SFC	MER	BR	GEN	BT	ACC
Separation Angle (°)	Over-stride	−7.89 * (26.7)	38.9 * (9.40)	17.5 (10.5)	10.7 (8.92)	11.2 ** (13.5)	33.4 (8.04)	13.2 (2.16)
	Under-stride	−16.1 (30.0)	25.3 (24.1)	20.0 (10.3)	10.9 (16.1)	−1.45 (11.2)	31.7 (4.89)	14.7 (3.17)
Proximal Plyometric Effect (Trunk_ω_/Pelvis_ω_)	Over-stride	1.53 (1.18)	0.77 (0.39)	1.81 (0.51)	2.75 * (0.28)	1.34 (1.76)	1.53 * (0.69)	2.23 * (0.41)
	Under-stride	1.36 (2.10)	0.53 (0.34)	1.75 (0.73)	3.37 (0.22)	1.53 (1.96)	1.05 (0.40)	2.40 (0.60)

Mean (SD) for pelvic–trunk separation angles and proximal plyometric effect at normalized events and phases. Significant differences indicated (*p* < 0.001) ** and (*p* < 0.05) *. PKH, Peak Knee Height; SFC, Stride Foot Contact; MER, Maximal External Rotation; BR, Ball Release; GEN, Generation Phase; BT, Brace-Transfer Phase; ACC, Acceleration Phase. Pelvic separation behind trunk (−)/pelvic separation ahead of trunk (+) and proximal plyometric effect following peak separation. US indicated greater (−) separation at PKH. Greater (+) separation was observed for OS during GEN and at SFC.

**Table 5:** The timing of pelvic–trunk separation and the proximal plyometric effect in the pitching cycle.

		Angle	% Time			Ratio	% Time
Peak Separation (°)	Over-stride	41.2 (5.38)	84.1 (6.13)	Peak Plyometric Ratio (Trunk_ω_/Pelvis_ω_)	Over-stride	2.75 * (0.28)	98.0 (1.25)
	Under-stride	37.3 (7.83)	85.4 (7.84)		Under-stride	3.37 (0.12)	98.6 (1.15)

The timing of mean (SD) pelvic–trunk separation angles and the proximal plyometric effect relative to the normalized pitching cycle; significant differences indicated and (*p* < 0.05) *.

## Data Availability

Data is unavailable due to privacy restrictions.

## Abbreviations

The following abbreviations are used in this manuscript:

PTS	Axial pelvic–trunk separation	MER	Maximal external rotation
PPE	Proximal plyometric effect	BR	Ball release
OS	Over-stride	GEN	Generation phase
US	Under-stride	BT	Brace-Transfer phase
PKH	Peak knee height	ACC	Acceleration phase
SFC	Stride foot contact		
